# A Novel Proteomics-Based Clinical Diagnostics Technology Identifies Heterogeneity in Activated Signaling Pathways in Gastric Cancers

**DOI:** 10.1371/journal.pone.0054644

**Published:** 2013-01-25

**Authors:** Jeeyun Lee, Sung Kim, Phillip Kim, Xinjun Liu, Tani Lee, Kyoung-Mee Kim, In-Gu Do, Joon Oh Park, Se Hoon Park, Jiryeon Jang, Nicholas Hoe, Gulia Harvie, Anne Kuller, Anjali Jain, Gary Meyer, Glen Leesman, Young Suk Park, Min Gew Choi, Tae Sung Sohn, Jae Moon Bae, Ho Yeong Lim, Sharat Singh, Won Ki Kang

**Affiliations:** 1 Division of Hematology-Oncology, Department of Medicine, Samsung Medical Center Sungkyunkwan University School of Medicine, Seoul, Korea; 2 Department of Surgery, Samsung Medical Center Sungkyunkwan University School of Medicine, Seoul, Korea; 3 Department of Pathology, Samsung Medical Center Sungkyunkwan University School of Medicine, Seoul, Korea; 4 Research and Development, Oncology, Prometheus Laboratories, San Diego, California, United States of America; 5 Samsung Cancer Research Institute, Samsung Medical Center, Seoul, Korea; The Chinese University of Hong Kong, Hong Kong

## Abstract

**Purpose:**

The aim of this study was to utilize the proteomics-based Collaborative Enzyme Enhanced Reactive (CEER) immunoassay to investigate protein tyrosine phosphorylations as diagnostic markers in gastric cancers (GCs).

**Experimental Design:**

Protein lysates from fresh-frozen 434 advanced stage GCs were analyzed for phosphorylation of HER1, HER2, p95HER2, HER3, cMET, IGF1R and PI3K. The pathway activation patterns were segregated based on the tumor HER2 status. Hierarchical clustering was utilized to determine pathway coactivations in GCs. Prognostic value of pathway activation patterns was determined by correlating disease-free survival times of the various GC subgroups using Kaplan-Meier survival analysis. CEER was also used to determine the presence of tyrosine phosphorylated signaling cascades in circulating tumor cells (CTCs) and ascites tumor cells (ATCs).

**Results:**

Utilizing a novel diagnostics immunoassay, CEER, we demonstrate the presence of p95HER2 and concomitantly activated signaling pathways in GC tumor tissues, CTCs and ATCs isolated from GC patients for the first time. p95HER2 is expressed in ∼77% of HER2(+) GCs. Approximately 54% of GCs have an activated HER1, HER2, HER3, cMET or IGF1R and demonstrate a poorer prognosis than those where these receptor tyrosine kinases (RTKs) are not activated. Hierarchical clustering of RTKs reveals co-clustering of phosphorylated HER1:cMET, HER2:HER3 and IGF1R-PI3K. Coactivation of HER1 with cMET renders GCs with a shorter disease-free survival as compared to only cMET activated GCs.

**Conclusions:**

Our study highlights the utility of a novel companion diagnostics technology, CEER that has strong implications for drug development and therapeutic monitoring. CEER is used to provide an increased understanding of activated signaling pathways in advanced GCs that can significantly improve their clinical management through accurate patient selection for targeted therapeutics.

## Introduction

Molecularly targeted agents have accelerated the field of oncology. Given that approximately half of the tyrosine kinase component of the human kinome is implicated in human cancers, it is not surprising that a large proportion of the current therapeutics target various kinases [1], [2]. Coactivation of receptor tyrosine kinases (RTKs) has been observed in subsets of multiple cancers such as glioblastoma multiforme [3], breast cancer [2], [4], [5], [6], [7], lung cancer [8], [9], head and neck cancer [7] and gastric cancer [4], [5], [6] thus implicating them as necessary for tumor progression and survival. These observations provide sufficient clinical rationale for screening activated RTKs in cancers that could then guide therapeutic targeting and potentially provide knowledge for rational combinatorial therapeutic strategies. However, simply analyzing the expression or activation status of a single RTK that may be the specific target protein for a particular therapeutic is insufficient for selecting patients as often times patients fail to respond to therapies despite the expression of the target. Several potential issues could be responsible for such a lack of therapeutic benefit from targeted agents, e.g., 1) concomitant activation of parallel or alternate pathways which bypass the originally targeted protein(s), and 2) continuous alteration in molecular and pathologic features of neoplastic tissues during cancer progression. Therefore, it would be ideal to have a companion diagnostic tool that could be applied on limited amounts of clinical specimens to capture the comprehensive complexity of phosphorylated signaling networks in the neoplastic tissue in addition to the direct target RTK protein to monitor the “evolving” disease.

Targeted phosphorylation analysis of clinical samples has been hampered due to the lack of readily usable clinical assays that are sensitive enough to function on limited quantities of clinical tissues. We have previously reported the development of a novel proximity-based immunoassay, Collaborative Enzyme Enhanced Reactive-immunoassay (CEER; Figure S1) [6], which is suitable for analyzing both the total expression and activation status of protein signaling cascades. CEER can be performed in a multiplexed fashion directly on clinical samples that may be available in limited amounts. The CEER assay utilizes the formation of a unique immuno-complex requiring the co-localization of two detector enzyme-conjugated-antibodies once the target proteins have been captured on a microarray. This format enables the efficient and sensitive detection of RTKs as well as the downstream pathway proteins down to the single cell level (a sensitivity of about 100 zeptomoles). In this study we have utilized the CEER assay to study the signaling pathway complexity in gastric cancers (GC).

GCs are the leading cause of cancer death worldwide with an incidence of 18.9/100,000 cases per year and a mortality rate of 14.7/100,000 per year [10] and are the most common malignancy in Korea [11]. Metastatic GC remains a therapeutic challenge for medical oncologists due to its poor prognosis. Currently, trastuzumab is the only active targeted agent that has proven to be efficacious for GC in a randomized phase III trial [12]. While activation of several RTKs has been reported in GCs, their impact on GC prognosis is unknown. This is important for the development and application of therapeutics for the clinical management of GCs.

In this study, we have used the multiplexed CEER platform to determine levels of activated RTKs (HER1, HER2, truncated variants of HER2, i.e., p95HER2, HER3, cMET, PI3K, and IGF1R) in 434 fresh frozen GC tissues and attempted to categorize GC patients into potential subgroups based on their protein pathway activation patterns. We have observed multiple signal protein activations in subgroups of GC tumors that correlate with disease-free survival (DFS) in these patients. Therefore, we hypothesize that redundant pathway activation inputs could lead to residual downstream signaling in GCs, thus limiting the anti-tumor efficacy of monotherapies targeted against single RTKs. Finally, we have developed a potentially powerful methodology which may enable clinicians to monitor baseline and evolving alterations in tumor RTK activation profiles over the course of a treatment using circulating tumor cells (CTCs) and malignant cells isolated from peritoneal fluid (ascites tumor cells or ATCs). Taken together, our study provides evidence for concomitant RTK activation in GC human tissues besides establishing CEER as an efficient clinical tool to diagnose and monitor RTK activation during GC treatment or disease progression.

## Materials and Methods

### Patient Cohort and Tissue Specimen Procurement

The study was conducted after approval from the Samsung Medical Center Institutional Review Board (SMC IRB) for informed consent waiver using archival tissues with retrospective clinical data. The primary tumor samples were all collected from Samsung Medical Center. All patients underwent gastrectomy with radical lymph node dissection with curative intent. From March 2001 to February 2005, 447 fresh frozen tissues obtained from 434 patients were collected from surgically resected primary gastric tumors and were available for final analysis. All fresh frozen tissues were collected (within <30 minutes) at the surgical field and were immediately snap frozen and stored in liquid nitrogen until later use. Tumor specimens were confirmed for the presence of >70% tumor area by the pathologist. For the analysis, small pieces of frozen tissues (10 µm section X 3) were prepared using prechilled razor blade and lysed in 100 µL of lysis buffer. The resulting lysates were stored at −80°C until subsequent analysis.

### Histopathology Review and HER2 status determination

All available H&E-stained slides were centrally reviewed by two pathologists (I.D., K.K.M) at the experimental pathology core of the Samsung Cancer Research Institute. All tumor specimens were from surgically resected primary gastric tumors. HER2 status was determined by IHC and FISH. IHC analysis was performed using the HercepTest™ (Dako, Glostrup, Denmark). HER2 protein expression levels were scored as 0 to 3+, according to the consensus panel recommendations on HER2 scoring for GC [13], [14]. For FISH, PathVysion HER2 DNA probe kit (Abbott, Des Plaines, IL) was used. Ariol image analysis system was used to count the hybridization signals (Genetix, San Jose, USA). All samples with ratios of HER2/CEP17 between 1.8 and 2.2 were scored manually by counting more than 60 non-overlapping cells. Ratios of >2.2, 1.8 to 2.2, or <1.8 were classified as positive, equivocal, or negative for amplification, respectively. Chromosomal 17 polysomy was defined as a CEP17 signal which had more than six copies on average per cell. Of the 447 specimens, 434 specimens were included in the final analysis.

### Collaborative Enzyme Enhanced Reactive-immunoassay (CEER)

CEER slides were printed, incubated with GC lysates and processed as described earlier [6]. Slides were scanned at four photomultiplier (PMT) gain settings to increase the effective dynamic range. Data were fit to a five-parameter equation derived as a function of capture-antibody concentration and PMT and presented in computed units (CU).

#### Multiplexed-microarray printing

Capture antibodies were printed on nitrocellulose-coated glass slides (ONCYTE®, Grace Bio-Labs) using non-contact printers (Nanoplotter, GeSiM). The spot diameter was approximately 175 µm. Slides were kept in a desiccated chamber at 4°C. Approximately 500 pL of capture Abs were printed in triplicate at serial dilution concentrations of 1 mg/mL, 0.5 mg/mL, and 0.25 mg/mL. Purified mouse-IgGs served as negative controls. Immuno-array slide configurations are shown in Figure S1.

#### Antibody conjugation and purification

Target-specific antibodies (mouse monoclonal against specific epitopes on human signal transduction proteins) and the corresponding detector enzymes, glucose oxidase (GO) or horseradish peroxidase (HRP), were activated with bi-functional cross-linker, succinimidyl-4-(N-maleimidomethyl) cyclohexane-1-carboxylate (SMCC), and coupled yielding antibody-enzyme conjugates. Conjugates were purified by HPLC. Antibody activities in the purified conjugates were determined by competition ELISA and the post-conjugation enzyme activities were detected by functional assays specific for each detector enzyme.

#### CEER assays

Immuno-microarray slides were rinsed 2X with TBST (50 mM Tris/150 mM NaCl/0.1% Tween-20, pH 7.2–7.4), blocked with 80 µL Whatman Blocking Buffer for 1 hr at RT, then washed 2X with TBST. Serially diluted lysate controls or samples in 80 µL dilution buffer (2% BSA/0.1% TritonX-100/TBS, pH 7.2–7.4) were added to designated sub-arrays on slides and incubated for 1 hour at RT. Slides were washed 4X (3 min. each), and detector Abs were added in 80 µL of reaction buffer and incubated for 2 hours at RT. After washing slides with TBST to remove unbound detector Abs, 80 µL of biotin-tyramide solution (5 µg/ml in 50 mM glucose/PBS) prepared from 400 µg/mL in ethanol solution (Perkin-Elmer Life Science) was added and incubated for 15 min in dark. GO/HRP-mediated tyramide signal amplification process was terminated by washing with TBST 4X, 3 min each. Local deposition of biotin-tyramide was detected by incubation with streptavidin (SA)-Alexa647 (Invitrogen) at 0.5 µg/mL in 2% BSA/0.1% Triton/TBS for 40 min. Upon completion of incubation, slides were washed 4X with TBST, dried and kept in dark until they were imaged via a microarray scanner.

For CEER data analysis, slides were scanned at four PMT gain settings to increase the effective dynamic range. Background-corrected signal intensities were averaged for spots printed in triplicate. Several criteria were used to filter data for further analyses, including limits on spot footprints, coefficient of variation for spot replicates, and overall pad background. For each assay, a standard curve was generated from serially diluted control cell lysates prepared from cell lines with well-characterized signal transduction proteins. Data were fit to a five-parameter equation derived as a function of capture-antibody concentration and PMT. The standard curve for each marker was fit as a function of log signal intensity, measured as relative fluorescence unit (RFU) vs. log concentration of cell lysates and referenced to the standard cell lines. For example, standard curve of serially diluted cell lysates prepared from BT474 was used to normalize HER2 expression and the degree of phosphorylation in each sample (Figure S2). Hence, a sample with 1 CU of HER2 expression has the RFU value equivalent to RFU value of 1 standard reference BT474 cell. As reference cells have 1∼2×10^6^ HER1 or HER2 receptors per cell with approximately 10% phosphorylated receptors, 1 CU represents the expression of 1∼2×10^6^ RTKs or 1∼2×10^5^ phosphorylated RTKs [6]. The limit of detection (LOD) value by CU was determined to be less than 1 CU for both expression and activation of HER2. Individual predictions from each dilution and gain were averaged into a single, final prediction.

### Truncated HER2 Enrichment

Full-length p185-HER2 receptors were depleted from tumor tissue lysates using magnetic-bead coupled antibodies specific to extracellular domain (ECD) of HER2 as shown in Figure S3. Resulting p185-HER2 depleted lysates, which contained enriched truncated HER2 (t-HER2) receptor proteins lacking the ECD, were used for subsequent quantification of t-HER2 expression and phosphorylation using the respective CEER assays. A cut off value of 500 CU was used to score for p95HER2 positivity per 20 µg of tissue analyzed. The total p95HER2 assay readout was relative to the signals generated from the standard curves generated using a control cell lysate from the BT474 breast cancer cell line.

### Cell culture and reagents

CEER assay control cell lines, MDA-MB-468, T47D, HCC827 and BT474 cells with varying degrees of ErbB-RTK expression were obtained from ATCC and grown at 37°C in 5% CO_2_ - for MDA-MB-468 (Dulbecco's minimal essential medium (DMEM)+10% FBS), BT474 (DMEM+10% FBS), and HCC827 and T47D (RPMI 1640+10% FBS, 0.2 U/ml bovine insulin). Cells were counted and washed with 1× PBS before growth factor stimulation. MDA-MB-468 cells were stimulated with 100 nM epidermal growth factor (EGF) or transforming growth factor a (TGFα), T47D cells were stimulated with 20 nM heregulin β (HRGβ) or 100 ng/mL Insulin-like Growth factor-1 (IGF1) in serum-free growth media for 5 or 15 min. Stimulated cells were washed with 1× PBS and then lysed (lysis buffer: 50 mM Tris, pH 7.4, 150 mM NaCl, 1% Trition X-100 and 2 mM Na_3_VO_4_) and kept on ice for 30 min before taking the supernatant for a subsequent assay or kept at −80°C.

### CEER marker determination and heat map clustering

Positivity for a given biomarker was defined as greater than the third quartile for an individual biomarker in the patient population of this study. Patients were ranked based on the CU value from the CEER assay for a given biomarker. Those patients greater than the third quartile cutoff were considered at highest risk to have elevated biomarker levels and therefore potentially activated signal pathways. Subsequent survival analysis was done to assess the predicative quality of these cutoffs.

The biomarker profile of the tumor samples was represented by a heat map. Each cell was colorized based on the decile rank of the activation of that marker. Each marker was ranked by deciles, represented by a distinct shade, with the scale indicating the color. Both the row (patient) and column (marker) clustering was shown. The complete linkage algorithm was used to obtain a hierarchical cluster (or dendrogram) by sequentially grouping the most correlated observations using the hclust function in R, called by the heatmap.2 function available with the gplots library of the statistical environment R: A Language and Environment for Statistical Computing (http://www.r-project.org/). Subgroups of markers were defined by the clustering that allowed comparisons of marker profiles for patients.

### CTC and ATC isolation

For CTC evaluation, 7.5 ml of blood samples were drawn into 10-ml evacuated ethylenediamine tetraacetic acid (EDTA) tubes. The CellSearch System (Veridex) was used for immuno-magnetic CTC isolation according to the protocol previously described [6] using ferrofluids conjugated to Ab against epithelial cell adhesion molecule. For ATC evaluation, cellular contents were enriched from 50 to 100 mL of ascites fluid by centrifugation and immuno-magnetic tumor cell isolation was performed in a similar fashion. Multiple sets of ATCs were treated with cocktails of growth factors (EGF, HRG, HGF and IGF1) with or without lapatinib and PHA-665,752 inhibitor combination.

## Results and Discussions

### Patient characteristics

Characteristics of 434 GC patients are provided in Table 1. All patients received gastrectomies with D2 lymph node dissection with 242 (55.8%) patients receiving subtotal gastrectomies. According to AJCC 2002 staging system, 86 patients had pathologic stage I, 116 had stage II, 126 had stage III and 106 had stage IV (35 patients with metastatic M1) GC. At the time of analysis, 226 patients were dead and 237 patients had documented recurrence. 70 patients had signet ring cell carcinoma. The 5-year overall and disease-free survival rates were 52.4% and 50.0%, respectively. All primary GC tissues were procured at the time of surgery and immediately snap frozen for future molecular analysis.

**Table 1:** Patient characteristics.

Characteristics	N = 434
Age (yrs)	
Median, range	61, 26–87
Sex	
Male	285 (65.7%)
Female	149 (34.3%)
Type of gastrectomy	
Subtotal gastrectomy	242 (55.8%)
Total gastrectomy	192 (44.2%)
Location of tumor	
Cardia	12 (2.8%)
Body	142 (32.7%)
Antrum	280 (64.5%)
Grade	
Well to moderately differentiated tubular	138 (31.8%)
Poorly differentiated tubular	186 (42.9%)
Signet ring cell	70 (16.1%)
Mucinous	32 (7.4%)
Papillary	2 (0.5%)
Hepatoid	2 (0.5%)
Others	4 (0.9%)
Lauren type (N = 397)	
Intestinal	154 (38.8%)
Diffuse	225 (56.7%)
Mixed	18 (4.5%)
Lymphovascular invasion	
Present/Identified	256 (59%)
Not Present/Not Identified	178 (41.0%)
AJCC stage (2002)	
I	86 (198.8%)
II	116 (26.7%)
III	126 (29.0%)
IV	106 (24.4%)

Patient characteristics of the gastric cancer clinical sample set.

### CEER-based assays reveal heterogeneity in HER2 expression and presence of HER2 truncated variant, p95HER2, in HER2(+) gastric cancers

We have previously reported the development of immuno-microarray based CEER assays [6], [15]. We used the CEER-based HER2 assay to investigate the HER2 status of the HER2(+) and HER2(−) samples in our GC patient cohort that were segregated based on standard IHC HercepTest/FISH analysis. The advantage of CEER-based assays is their higher sensitivity and specificity as compared to IHC-based assays [6]. Based on current HER2(+) definitions for GCs [13], [14], i.e., samples with a HER2-IHC score of 3+ or 2+ and a positive *HER2* gene amplification status, 50 of 434 (11.5%) samples in our GC patient cohort were HER2(+) by IHC HercepTest/FISH analysis (Table S1). In contrast, according to the IHC guidelines specific for breast cancers, only 78% (39/50) of these patients were HER2(+) (Table S1) indicating the differences in scoring criteria for HER2 positivity between gastric and breast cancers. A majority of the HER2(+) GCs (36 out of 50 samples (72%)) were of the intestinal subtype rather than the diffuse or mixed type GCs in agreement with published reports [13], [16], [17] .

**Figure 1:**
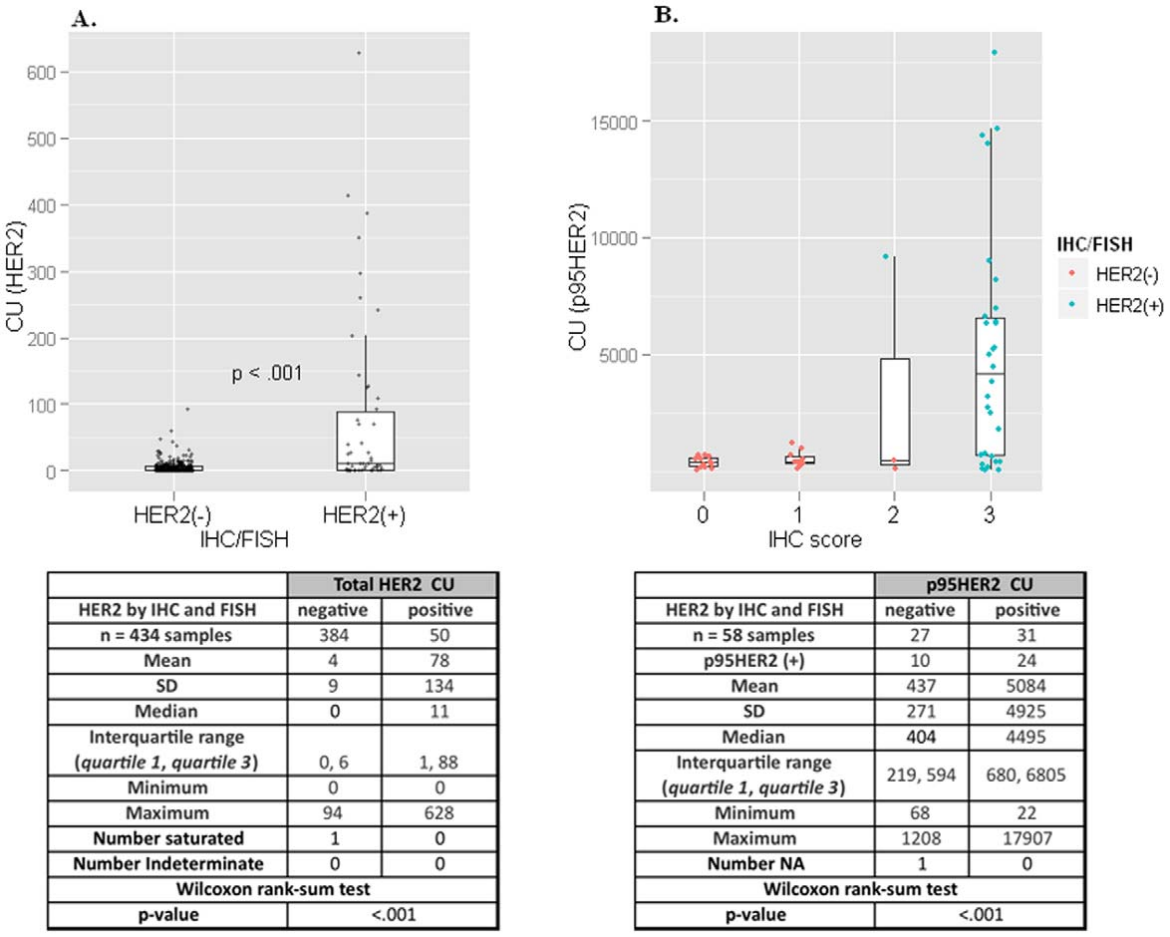
HER2 and p95HER2 expression in gastric cancers. (A) CU distribution for HER2 expression of GC samples at 0.25 µg lysate. The *x*-axis represents the IHC/FISH status, and the *y*-axis represents the CU values from CEER assay as determined from a BT474 standard curve. Separation is illustrated between the two groups with a median of 0 for the IHC/FISH negative population (384 of 434) compared to a median of 11 for the IHC/FISH positive population (50 of 434). One saturated sample, above the limit of quantitation and indicated as ‘Number saturated’ in the corresponding table, is not shown. Boxes represent the interquartile range, with the 75th percentile at the top and the 25th percentile at the bottom. The line in the middle of the box represents the median. Whiskers extend to the highest and lowest value within 1.5 times the interquartile range. *P* value<.001 was determined by Wilcoxon signed-rank test. (B) CU distribution for p95HER2 in a subset of the tumor samples (58 of 434) at 20 µg lysate. The *x*-axis represents the IHC status, and the *y*-axis represents the CU values from CEER assay for p95. Full length HER2 was removed by immuno-depletion prior to the assay. The data points are colored based on the HER2 status by IHC and FISH. As shown, one data point with an IHC of 2 was determined to be positive by FISH analysis. Of the 34 samples determined to be positive for p95 by CEER, 24 (71%) of them were HER2(+) by IHC/FISH. p95HER2 expression could not be determined in one sample which is indicated as ‘Number NA’ in the corresponding table.

CEER-based HER2 assay demonstrated substantially higher HER2 expression levels in IHC/FISH HER2(+) tumors as compared to those in the HER2(−) tumors (with a mean value of 77.9 CU vs. 4.3 CU, p-value 8.18E-10) as shown in Figure 1A. The CEER-based HER2 data is presented in CU, a standard functional unit based on cell line controls with known HER2 expression [6], that allows comparison of HER2 expression across samples. Only 32/50 or 64% of IHC/FISH HER2(+) GCs demonstrated HER2 expression by CEER and there was a significant level of heterogeneity in HER2 expression in these samples as revealed by the quantitative CEER readouts. Heterogeneity in HER2 expression may explain the reason for only a moderate degree of concordance (87.5%) between the IHC and FISH readouts for HER2 in the ToGA trial [12]. This discrepancy would directly affect the outcome of HER2-targeted therapeutics such as trastuzumab in GCs. Moreover, due to the high sensitivity of the CEER assay, it was noticed that ∼20% of the IHC/FISH HER2(−) GCs still expressed total HER2 albeit at distinctly lower levels than the HER2(+) patient population.

Using the CEER-based p95HER2 assay platform, truncated forms of HER2 were specifically detected, in addition to full length HER2, in GCs for the first time. p95HER2 expression was analyzed in 31/50 HER2(+) samples and 27/384 HER2(−) samples) that demonstrated a significant full length HER2 expression by CEER (Figure 1B). The incidence of p95HER2 in HER2(+) GCs was ∼77% (in 24/31 samples) (Table S1). Similar to full length HER2 expression, the majority of p95HER2 expression (79% of p95HER2 expressed in HER2(+)) was detected in intestinal type GCs. p95HER2 expression was also observed in a small percentage of HER2(−) GCs (37% or 10/27 samples) that demonstrated full length HER2 expression. However, the average p95HER2 expression in HER2(+) GC samples was significantly higher than that observed in HER2(−) GCs (5083.6 CU in HER2(+) vs 437.0 CU in HER2(−), p-value = 1.27e-05). p95HER2 may contribute to trastuzumab non-responsiveness in GC tumors as it does in 70–80% of HER2 overexpressing breast cancers [18]. Trastuzumab plus standard chemotherapy rendered an overall response of only 47% in HER2(+) GCs in the ToGA trial [12] indicating existence of possible trastuzumab resistance mechanisms and our inability to accurately select trastuzumab responders. In order to clinically validate p95HER2 expression with trastuzumab resistance, which has been controversial especially due to the recent conflicting findings from the GeparQuattro breast cancer studies [19], rigorous p95HER2 assay refinement in terms of clinically-relevant diagnostic assay cut-off determinations and applicability in clinical settings is required. A validation of p95HER2 expression is planned in a phase II neoadjuvant lapatinib plus chemotherapy clinical trial in GC patients as several studies suggest the use of lapatinib in p95HER2(+) cancers [20], [21].

Taken together, our data clearly define the HER2 status in GCs and demonstrate the utility of CEER-based HER2 diagnostics in GC patient samples for determining their accurate full length and truncated HER2 expression. The development of such diagnostics will strongly impact the accurate selection of HER2(+) GCs that are responders to trastuzumab and other HER2 targeting agents. We have previously reported the use of CEER-based HER2 diagnostics in breast cancer patients [6], [15] that demonstrated a higher sensitivity and specificity as compared to IHC-based diagnostics.

### Gastric cancers demonstrate concomitant activation of signaling pathways

**Figure 2:**
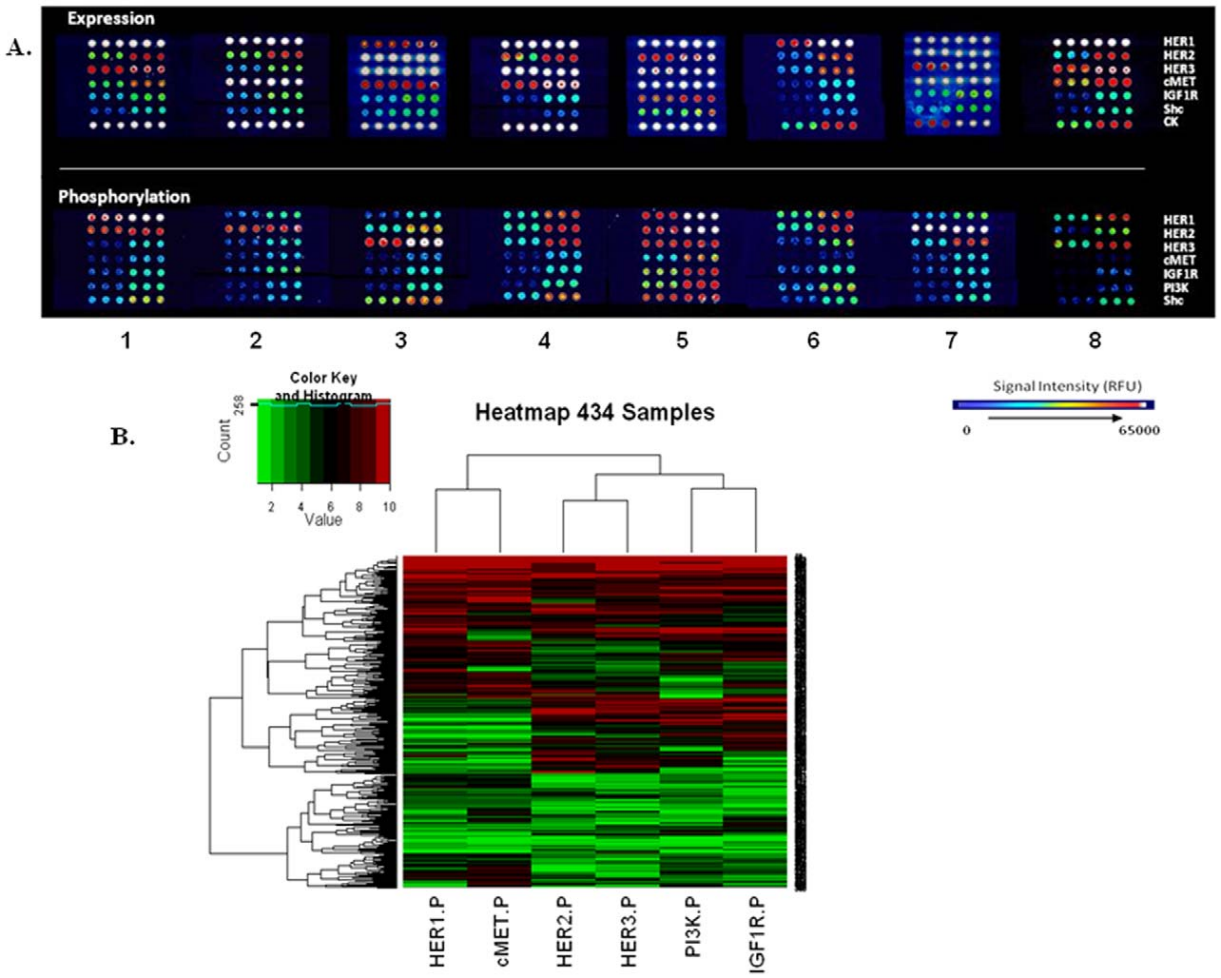
Profiling of phosphorylated markers in gastric cancers. (A) Representative immuno-array images for pathway profiling of indicated signal transduction proteins. Array signal intensity ranges from black/dark blue (low) to red/white (high/saturation). (B) Heat map and hierarchical clustering of the 434 samples based on CU values from CEER assay for phosphorylated markers measured at 10 µ g lysate concentration. Each column represents a marker and each row represents a patient sample. Relative levels of phosphorylation are depicted with a color scale where red represents the highest level of activation and green represents the lowest level. The CU values for each marker (column) were ranked by deciles. Jitter, between 0 and 0.1, was added to each biomarker CU value to create equally sized bins. Row and column dendrogram show the result of the hierarchical clustering calculation.

We used the CEER assay directly on GC samples to assess the presence of total and phosphorylated (activated) forms of several signaling molecules that are known drug targets: these included several RTKs (HER1, HER2, HER3, cMET, IGF1R) and PI3K. Representative images of multiplexed CEER pathway-arrays from eight different samples are shown in Figure 2A. These images clearly demonstrate the heterogeneity in activated pathway signatures that is prevalent in GCs. For example, both HER1/HER2 are coactivated in samples 1 and 6, whereas only HER2 is activated in sample 2 although HER1, HER2 and cMET are expressed to high levels. Sample 5 demonstrates activation of all 5 analyzed RTKs including the downstream PI3K and Shc pathways. The following section describes the signaling pathway heterogeneity observed in our GC patient cohort as determined by the CEER assays.

Tumors from 202 patients (46.5%) had no detectable activation of the RTKs tested in our study. Pathway activation patterns for the rest of the tumors, as depicted in a pathway clustering analysis (Figure 2B), varied widely with some GCs demonstrating concomitant activation of multiple RTKs such as HER2/HER3, HER1/2/3, HER1/3/cMET or HER3/cMET while others were phosphorylated only on a single protein. The tumor content of all the analyzed samples was >70% based on their histological examination. However, the pan-cytokeratin (pan-CK) expression was distinctly variable suggesting heterogeneity in the epithelial content of GCs. Based on this profiling, we categorized the 434 GC sample cohort according to their RTK activation signatures and HER2 status.

#### HER axis in gastric cancers (Table 2 & Table S1)

**Table 2:** Distribution of activated HER kinase axis members in GCs.

CEER MARKER	HISTOTYPE	HER2(+) (n = 50)	HER2(−) (n = 384)	Sample % based on histotype
**pHER1**	Intestinal (n = 154)	7	31	**24.7%**
	Diffuse (n = 225)	4	54	**25.8%**
	Mixed (n = 18)	2	1	**16.7%**
	ND (n = 37)	0	10	**27.0%**
% based on HER2 status		**26.0%**	**25.0%**	
**pHER2**	Intestinal (n = 154)	22	33	**35.7%**
	Diffuse (n = 225)	2	41	**19.1%**
	Mixed (n = 18)	1	5	**33.3%**
	ND (n = 37)	0	5	**13.5%**
% based on HER2 status		**50.0%**	**21.9%**	
**pHER3**	Intestinal (n = 154)	13	33	**29.9%**
	Diffuse (n = 225)	3	46	**21.8%**
	Mixed (n = 18)	2	3	**27.8%**
	ND (n = 37)	0	9	**24.3%**
% based on HER2 status		**36.0%**	**23.7%**	
**pHER1+pHER2**	Intestinal (n = 154)	6	23	**18.8%**
	Diffuse (n = 225)	1	27	**12.4%**
	Mixed (n = 18)	1	1	**11.1%**
	ND (n = 37)	0	5	**13.5%**
% based on HER2 status		**16.0%**	**14.6%**	
**pHER1+pHER3**	Intestinal (n = 154)	2	19	**13.6%**
	Diffuse (n = 225)	1	31	**14.2%**
	Mixed (n = 18)	2	1	**16.7%**
	ND (n = 37)	0	6	**16.2%**
% based on HER2 status		**10.0%**	**14.8%**	
**pHER2+pHER3**	Intestinal (n = 154)	11	22	**21.4%**
	Diffuse (n = 225)	1	28	**12.9%**
	Mixed (n = 18)	1	3	**22.2%**
	ND (n = 37)	0	4	**10.8%**
% based on HER2 status		**26.0%**	**14.8%**	
**pHER1+pHER2+pHER3**	Intestinal (n = 154)	2	17	**12.3%**
	Diffuse (n = 225)	1	22	**10.2%**
	Mixed (n = 18)	1	1	**11.1%**
	ND (n = 37)	0	4	**10.8%**
% based on HER2 status		**8.0%**	**11.5%**	

Distribution of samples with respect to each activated HER kinase axis receptor member and its coactivation with other HER members. HER2 status and histological subtype of the samples is indicated. ND: not defined.

At least one receptor member of the HER axis was activated in 41% of the GC patients. HER2 phosphorylation was detected not only in 50% of HER2(+) GC patients but also in ∼22% of HER2(−) cancers in agreement with the observed total HER2 expression described earlier. 16/25 HER2(+) samples that demonstrated activated HER2 also expressed p95HER2. Likewise, HER3 was also phosphorylated in a higher percentage of HER2(+) GCs (36%) as compared to HER2(−) cancers (∼24%). However, phosphorylated HER1 did not have such a preference and was equivalently activated in both GC types (26% in HER2(+) and 25% in HER2(−)). Furthermore, most of the HER member activated GC tumors (∼64%) demonstrated a concomitant activation of other RTKs, i.e., cMET or IGF1R. Histologically, a majority of the HER2(+), intestinal type GCs expressed an activated HER2 (in 22/36 or ∼61%) followed by HER3 (in 13/36 or ∼36%) with an overall 36% of intestinal type GCs expressing an activated HER2 pathway. Overall, HER2 activation was more concentrated in intestinal type (55/154 or 35.7%) as compared to the diffuse-type cancers (43/225 or 19.1%). In contrast, activated HER1 was equally observed in both intestinal-type (38/154 or 24.7%) and diffuse type (58/225 or 25.8%) GCs. Activated HER3 was also equivalently present (29.9% and 21.8%) between both Lauren's classification subtypes.

As the signaling function of the HER kinase axis is dependent upon activated receptor dimerization, we looked at the various pairs of HER member coactivations. Coactivation of HER2 with HER3 was preferred in HER2(+) cancers (13/50 or 26%) with 7/13 HER2:HER3 activated GCs without a HER1 coactivation. Approximately 54% of the HER2:HER3 coactivated HER2(+) GCs coexpressed p95HER2. On the other hand, HER2(−) GCs did not demonstrate a preference for any specific HER kinase dimer pair with all three possible activated dimer pairs (HER1:HER2, HER1:HER3 and HER2:HER3) expressed in ∼15% each of HER2(−) samples. Triple activation of HER1/2/3 receptors was observed in 11.1% of GCs with a marginally higher distribution in HER2(−) cancers.

#### cMET activated gastric cancers (Table 3 & Table S1)

Similar to HER1, cMET phosphorylation was equivalently distributed between HER2(+) (22%) and HER2(−) (∼25%) GCs. Furthermore, activated cMET distribution based on the Lauren histotype was similar between intestinal (48/154 or 31.2%) and diffuse-type (55/225 or 24.4%) GCs.

Majority of cMET activated GC samples (∼71% or 77/108) demonstrated a concomitant activation of HER kinase receptor members. All samples with a phosphorylated cMET demonstrated a *cMET* amplification (data not shown). We investigated the preferred HER axis receptors that cross-talk with the cMET pathway. Of the 77 GC samples demonstrating a cMET coactivation with a HER member, 66 samples (∼86%) were with HER1. Overall too, cMET was preferentially coactivated with HER1 (15.2%) as compared to HER2 (10.1%) or HER3 (9.7%). These observations suggest a possible cross-talk between the cMET and HER1 signaling pathways in GCs. cMET activation never co-existed with activated HER3 unless HER1 and/or HER2 were also phosphorylated in the same sample. Approximately 7% of GC patients demonstrated a coactivation of all three HER axis receptor members with cMET. Activated cMET co-existed with p95HER2 expressing samples in 5/24 and 1/10 HER2(+) and HER2(−) GCs, respectively.

#### IGF1R activated gastric cancers (Table 4 & Table S1)

**Table 4:** Distribution of activated IGF1R kinase receptor in GCs.

CEER MARKER	HISTOTYPE	HER2(+) (n = 50)	HER2(−) (n = 384)	Sample % based on histotype
**pIGF1R**	Intestinal (n = 154)	11	29	**26.0%**
	Diffuse (n = 225)	3	52	**24.4%**
	Mixed (n = 18)	1	4	**27.8%**
	ND (n = 37)	0	10	**27.0%**
% based on HER2 status		**30.0%**	**24.7%**	
**pIGF1R+pHER1**	Intestinal (n = 154)	2	15	**11.0%**
	Diffuse (n = 225)	2	30	**14.2%**
	Mixed (n = 18)	1	1	**11.1%**
	ND (n = 37)	0	7	**18.9%**
% based on HER2 status		**10.0%**	**13.8%**	
**pIGF1R+pHER2**	Intestinal (n = 154)	8	18	**16.9%**
	Diffuse (n = 225)	1	22	**10.2%**
	Mixed (n = 18)	0	1	**5.6%**
	ND (n = 37)	0	4	**10.8%**
% based on HER2 status		**18.0%**	**11.7%**	
**pIGF1R+pHER3**	Intestinal (n = 154)	7	18	**16.2%**
	Diffuse (n = 225)	3	25	**12.4%**
	Mixed (n = 18)	1	1	**11.1%**
	ND (n = 37)	0	7	**18.9%**
% based on HER2 status		**22.0%**	**13.3%**	
**pIGF1R+pHER1+pHER2+pHER3**	Intestinal (n = 154)	2	9	**7.1%**
	Diffuse (n = 225)	1	18	**8.4%**
	Mixed (n = 18)	0	1	**5.6%**
	ND (n = 37)	0	3	**8.1%**
% based on HER2 status		**6.0%**	**8.1%**	
**pIGF1R+pMET**	Intestinal (n = 154)	1	16	**11.0%**
	Diffuse (n = 225)	1	28	**12.9%**
	Mixed (n = 18)	0	0	**0.0%**
	ND (n = 37)	0	4	**10.8%**
% based on HER2 status		**4.0%**	**12.5%**	
**pIGF1R+pHER1+pHER2+pHER3+pMET**	Intestinal (n = 154)	1	6	**4.5%**
	Diffuse (n = 225)	1	16	**7.6%**
	Mixed (n = 18)	0	0	**0.0%**
	ND (n = 37)	0	2	**10.8%**
% based on HER2 status		**4.0%**	**6.3%**	

Distribution of samples with respect to activated IGF1 receptor and its coactivation patterns with other RTKs. HER2 status and histological subtype of the samples is indicated. ND: not defined.

Similar to HER3 activation, the signaling pathway driven by the IGF1 receptor was active in a higher percentage of HER2(+) GCs (30%) than the HER2(−) GCs (24.7%). Furthermore, as observed with phosphorylated HER3, activated IGF1R was equivalently distributed between the intestinal (26%) and diffuse-type (∼24%) GCs. This distribution lead us to investigate if there was an IGF1R:HER3 coactivation in the GC patient cohort. IGF1R was indeed maximally coactivated with p-HER3 especially in HER2(+) GCs where 22% or 11/50 HER2(+) GCs demonstrated an IGF1R:HER3 coactivation. However, in HER2(−) GCs, percentage of IGF1R coactivation with HER3 (in 51/384 samples or 13.8%) was similar to IGF1R coactivation with HER1 (in 53/384 samples or 13.3%). IGF1R:HER2 coactivation (in 45/384 samples or 11.7%) followed close behind. Overall, 34/434 GC samples demonstrated coactivation of all members of the HER kinase axis with IGF1R of which 28 samples also demonstrated a cMET coactivation. However, cMET was rarely co-activated with IGF1R in the absence of a HER kinase receptor member coactivation. Furthermore, c-MET:IGF1R coactivations were primarily observed in HER2(−) GCs.

GC samples were hierarchically clustered based on their decile-based marker activation profiles (Figure 2B). The analysis demonstrated that cMET:HER1, HER2:HER3 and IGF1R:PI3K activation patterns were closely correlated with the cMET:HER1 cluster forming a distinct subset. Further analysis of signaling pathways resident downstream of the analyzed RTKs such as PI3K revealed that in the unselected GC population, PI3K activity was maximally observed with IGF1R coactivation (77/108 or 71.3%) followed by HER3 coactivation (71/108 or 65.7%). This is in agreement with the hierarchical clustering results as well as published reports further strengthening the validity of the CEER assay.

### GC patient segmentation based on CEER-based pathway activation profiles correlates with post-operative DFS

We investigated the impact of CEER-based phosphorylation profiling on clinical prognosis of GCs. DFS following curative surgery was analyzed after segregating the patients into groups based on the signaling pathway activation profiles of their tumor samples. DFS refers to time from surgery to definite recurrence or deaths.

**Figure 3:**
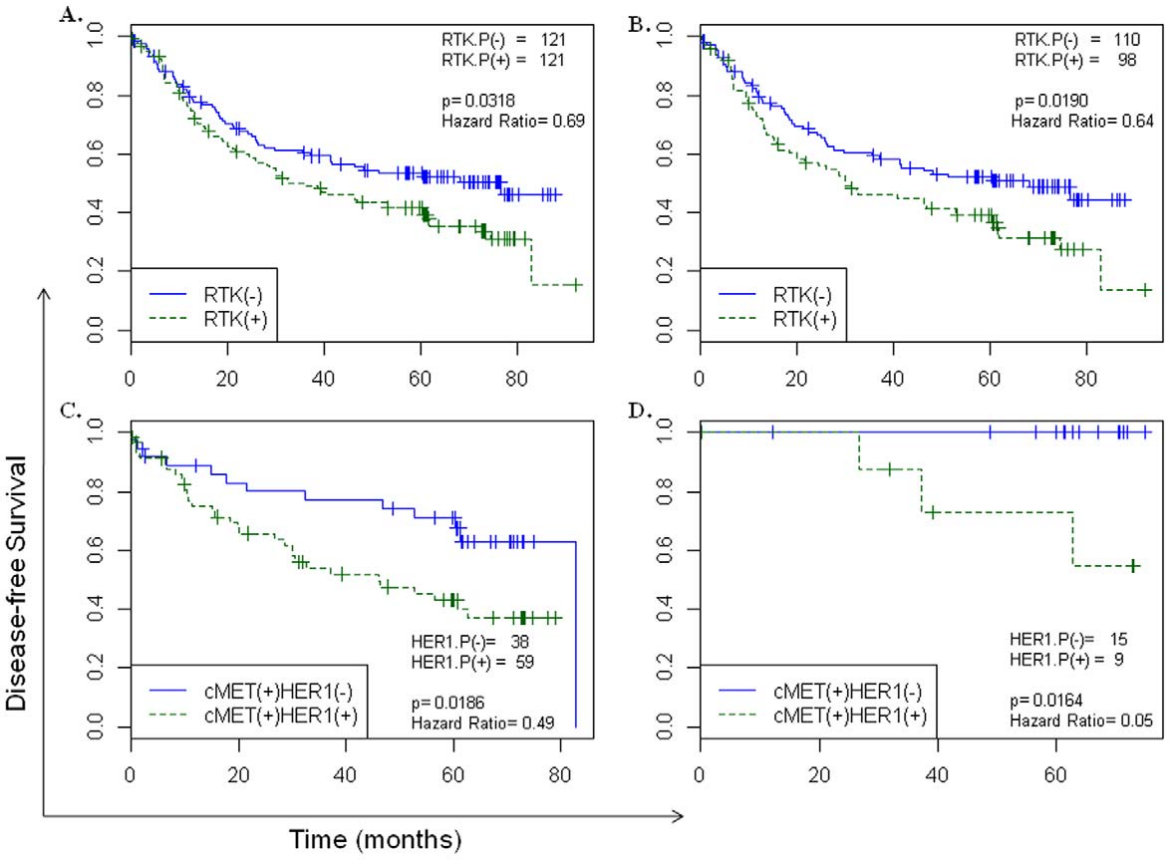
Disease-free survival differences in gastric cancers based on activated RTK profiling. (A & B) Disease free survival differences in GC samples of tumor stages II+III. The analysis compared the overall GC sample set (A) or only HER2(−) GC sample set (B) between cohorts with 0 RTK activation vs ≥1 RTK activation. Median survival of the two cohorts in all patients is 32.63 months (≥1 RTK activation) and 76.53 months (0 RTK activation) and in patients with HER2(−) GC is 30.10 months (≥1 RTK activation) and 68.13 months (0 RTK activation). (C & D) Disease free survival differences comparing HER1(−) cMET(+) vs HER1(+) cMET(+) cohorts in HER2(−) (C) or Stage I, HER2(−) (D) GC samples. Median survival of the two cohorts in HER2(−) patients is 46.17 months (HER1(+) cMET(+)) and 82.80 months (HER1(−) cMET(+)). Median survival times in patients with HER2(−) stage I GC are undefined for both cohorts. Sample numbers in each cohort, p-values and hazard ratios are indicated.

There was no significant difference in DFS between HER2(+) and HER2(−) GC groups. p95HER2 positive, HER2(+) samples indicated a worse survival trend as compared to p95HER2 negative, HER2(+) samples but these differences were not significant .We next analyzed the survival differences between patients with no RTK activations versus patients with ≥1 RTK activation. Stage II+III GC patients with ≥1 RTK activation demonstrated a considerably worse survival than those where none of the analyzed RTKs were activated (Figure 3A; Tumor stages II+III: 32.63 months for ≥1 RTK activation vs 76.53 months for 0 RTK activation (p = 0.0318, hazard ratio = 0.69)). Significant differences in median survival times were maintained in HER2(−) only samples as well (Figure 3B; Tumor stages II+III: 30.10 months for ≥1 RTK activation vs 68.13 months for 0 RTK activation (p = 0.0190, hazard ratio = 0.64)). These data clearly indicate that the RTKs analyzed in this study, in various combinations, influence DFS in GCs. An important observation from this analysis was that the activated status of the RTKs, but not simply their expression status, contributed towards a worse DFS post-curative surgery.

In an effort to explore the contribution of individual RTKs on DFS, we concentrated on the HER1 and cMET activated GC patients. The reasons for focusing on these RTKs were several. *cMET* is gene amplified in a significant number of GCs; however, single agent anti-MET inhibitors have failed to demonstrate a clinical benefit in these cancers [22]. On the contrary, cMET and HER1 combination therapy has demonstrated superior efficacy in preclinical models of GCs [23]. Using CEER, we observed coactivation of cMET and HER1 including their co-clustering in GC patients (Table 3A and Figure 2B). These observations suggested that the overall characteristics of p-HER1:p-cMET co-expressing GC samples may be distinctly different from samples that express p-cMET without an activated HER1. We compared DFS in p-HER1(−):p-cMET(+) and p-HER1(+):p-cMET(+) sample sets. There was a significant difference in median survival times of these cohorts in the tumor stage independent GC patient population (Figure S4; 46.17 months for p-HER1(+):p-cMET(+) vs 82.80 months for p-HER1(−):p-cMET(+) (p = 0.0184, hazard ratio = 0.51)). Similar results were observed in HER2(−) only GCs (Figure 3C). Subsequently, the p-HER1(−):p-cMET(+) and p-HER1(+):p-cMET(+) cohorts were analyzed in the HER2(−) GC subset in a tumor stage-adjusted manner. Coactivation of cMET and HER1 in stage I GCs showed a worse DFS when compared to stage I patients with cMET activation alone (Figure 3D). Our data strongly suggests that the p-HER1(+):p-cMET(+) GC samples have a poorer prognosis. For this reason, there maybe value in further characterizing GC patients based on their pathway activation profiles to identify candidates for more aggressive adjuvant treatment options post surgery.

**Table 3:** Distribution of activated cMET kinase receptor in GCs.

CEER MARKER	HISTOTYPE	HER2(+) (n = 50)	HER2(−) (n = 384)	Sample % based on histotype
**pMET**	Intestinal (n = 154)	9	39	**31.2%**
	Diffuse (n = 225)	2	53	**24.4%**
	Mixed (n = 18)	0	0	**0.0%**
	ND (n = 37)	0	5	**13.5%**
% based on HER2 status		**22.0%**	**25.3%**	
**pMET+pHER1**	Intestinal (n = 154)	5	19	**15.6%**
	Diffuse (n = 225)	2	35	**16.4%**
	Mixed (n = 18)	0	0	**0.0%**
	ND (n = 37)	0	5	**13.5%**
% based on HER2 status		**14.0%**	**15.4%**	
**pMET+pHER2**	Intestinal (n = 154)	7	16	**14.9%**
	Diffuse (n = 225)	1	18	**8.4%**
	Mixed (n = 18)	0	0	**0.0%**
	ND (n = 37)	0	2	**5.4%**
% based on HER2 status		**16.0%**	**9.4%**	
**pMET+pHER3**	Intestinal (n = 154)	3	11	**9.1%**
	Diffuse (n = 225)	1	25	**11.6%**
	Mixed (n = 18)	0	0	**0.0%**
	ND (n = 37)	0	3	**8.1%**
% based on HER2 status		**8.0%**	**10.2%**	
**pMET+pHER1+pHER2+pHER3**	Intestinal (n = 154)	3	8	**7.1%**
	Diffuse (n = 225)	1	17	**8.0%**
	Mixed (n = 18)	0	0	**0.0%**
	ND (n = 37)	0	2	**5.4%**
% based on HER2 status		**8.0%**	**7.0%**	

Distribution of samples with respect to activated cMET receptor and its coactivation patterns with other RTKs. HER2 status and histological subtype of the samples is indicated. ND: not defined.

### CEER can be utilized as a non-invasive diagnostic tool to interrogate pathway activation signatures in CTCs and ATCs

We used the CEER assays to evaluate the activated signaling pathways in CTCs and ATCs isolated from metastatic GC patients as it is almost impossible to obtain tumor specimens from such patients. The levels of HER2 expression and phosphorylation were determined in CTCs isolated from 105 metastatic GC patients [24]. Evaluable CTCs were found in 33% (35/105) of enrolled patients. Out of 35 patients, 7 patients (20%) demonstrated high HER2 over expression, 6 patients (17%) had moderate HER2 expression and 11 patients (31%) showed HER2 activation (phospho positive) with no HER2 over-expression. CEER-based pathway analysis demonstrated heterogeneity in activated RTK patterns in CTCs that was similar to the GC tumor specimens.

**Figure 4:**
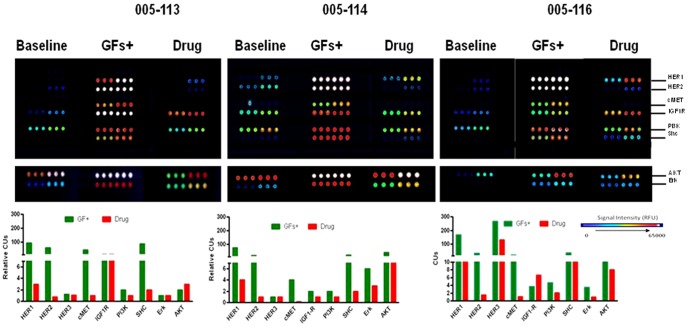
Profiling of phosphorylated markers in CTCs and ATCs from gastric cancer patients. RTK and downstream pathway profiling in ATCs isolated from 3 patients after ligand (EGF, Heregulin, HGF and IGF) stimulation with or without 2 µM inhibitor cocktail (lapatinib and PHA-665,752) or DMSO. Relative CU is defined as the ratio of CUs over baseline (no ligand or drug treatment).

Subsequently, we successfully isolated malignant cells from ascites fluid, another source of noninvasive cancer cells in GC where the yields are significantly greater than the CTCs. A drainage of 100 mL of ascites fluid yielded tumor cells that were several magnitudes higher (>1×10^3^ to 1×10^4^) than typical yields of CTCs from 7.5 ml of blood. Like the CTCs, heterogeneous RTK activation patterns were also seen in ATCs isolated from GC patients. We determined if the signaling pathways in ATCs would be responsive to *ex vivo* ligand and/or drug perturbations as this could potentially provide valuable information regarding the functionality and drug responsiveness of ATCs that may be indicative of the *in situ* cancers. Representative examples from three different GC patients are shown (Figure 4). Significant levels of p-HER1, p-HER2 and p-MET after ligand stimulation were observed in all three sets of ATCs suggesting that lapatinib and PHA-665,752 combination may benefit these patients. Indeed this drug combination was effective in decreasing phosphorylations of these targets albeit to varying degrees. Whether or not this treatment regimen would translate into a clinical benefit for such patients remains to be seen. Profiling of other RTKs indicated that HER3 and IGF1R were also activated to varying levels in specific samples. It is possible that these RTKs may influence the outcome of the tested therapeutics as they could not be efficiently inhibited by the inhibitor cocktail. In fact, one patient (005-116) demonstrated an increase in p-IGF1R with inhibitor treatment. An analysis of the downstream signaling molecules (PI3K, SHC, Erk and AKT) revealed heterogeneity in terms of their ligand-induced activation and drug treatment profiles. Clinical relevance of these observations needs to be evaluated and expanded in a clinical trial; nonetheless, these data clearly demonstrate an ATC-based non-invasive platform that can be readily utilized for the evaluation of signaling pathways using CEER assays before and after therapeutic treatments in GC patients.

## Conclusions

Our study describes the utility of a novel proteomics technology, CEER, for diagnosing and molecularly stratifying the complexity of gastric cancers. CEER can be directly performed on GC clinical specimens and surrogate tissues that can allow active management of GCs. GC is an exceedingly heterogeneous disease where the heterogeneity is recognized at multiple levels [25], [26], [27], [28], [29], [30], [31], [32], [33], [34] that significantly limit its prognosis in terms of recurrence and response to therapy. Using CEER, our study identifies yet another level of heterogeneity based on signaling pathway signatures that will directly affect the selection and outcome of targeted therapeutics in this cancer type.

Specifically, several key and novel observations were noted that would have significant implications in the clinical management of GCs:

1. In addition to demonstrating an increased understanding of HER2(+) GCs including the presence of p95HER2 that can allow better selection for HER2 targeted therapies, our study is the first to provide a molecular understanding of HER2(−) GCs that form the majority of all GCs and for which no approved targeted therapies are currently available. Approximately 20% of HER2(−) GCs expressed phosphorylated HER2 indicating that these GCs may also utilize HER2 signaling for tumor growth, and thus may potentially respond to HER2 targeting agents. Indeed, trastuzumab is beneficial in a fraction of HER2(−) breast cancer patients [35]. Furthermore, subsets of HER2(−) GCs may also benefit from anti-HER1, HER3, cMET or IGF1R therapeutics as these RTKs were activated in significant numbers of HER2(−) GCs. HER3 protein overexpression has been previously correlated with poor prognosis in GCs [36], [37].
2. A combination of targeted agents, rather than individual therapies, may be more effective in GCs. This hypothesis is generated by the observation that a significant number of GCs (48% of HER2(+) and ∼32% of HER2(−)) are driven by networks of concomitantly activated RTKs instead of single RTKs. Indeed, a phase II trial of the cMET inhibitor, foretinib (XL-880) monotherapy in unselected GC patients failed to demonstrate an objective response [22].
3. pMET:pHER1 coactivated GCs were identified as a distinct subset that demonstrate a poor prognosis and could benefit from simultaneous inhibition of both cMET and HER1 RTKs. Cross-talk between cMET and HER1 has been previously observed preclinically in GC and lung cancer cell lines [4], [5], [6], [38] with a superior efficacy from a simultaneous blockade of the two signaling pathways [4], [5], [6], [23].
4. Utilizing CEER, GCs could be non-invasively evaluated using CTCs and ATCs as demonstrated in the present study. This is especially useful for metastatic GCs where tumor re-biopsies are nearly impossible. Given the fact that not all metastatic GC patients will have CTCs available for analysis, ATC analysis will definitely expand the applicability and clinical feasibility of the phospho-RTK assay in such patients.

In summary, our study describes a novel technology that can be utilized to not only diagnose GCs using limited clinical specimens but also provides a way to dissect the complex GC biology. With a signaling pathway monitoring tool utilizing CTCs and/or body fluids, we may be able to rapidly identify the required combinatorial treatments for advanced GC patients targeting multiple signaling pathways.

## Supporting Information

Figure S1 Principle of the CEER assay. Schematic showing the principle of the CEER assay and array layout. (Figure reproduced from Kim et al., 2011)
(TIF)

Figure S2 Standard curve for total HER2 and phosphorylated HER2. Standard curve of serially diluted cell lysates prepared from BT474 was used to normalize HER2 expression and the degree of phosphorylation in each sample. Each curve was plotted as a function of log signal intensity, measured as relative fluorescence unit (RFU) vs. log concentration of cell lysates and referenced to the standard cell lines. Image shown at single PMT setting, but multiple PMT scanning extends the dynamic range for the quantitation. Each row of the image shows total HER2 (HER2-T) and phosphorylated HER2 (HER2-P) expression in increasing numbers of cells. As shown, three different increasing concentrations of the HER2 capture antibodies were printed in triplicate on each CEER array.
(TIF)

Figure S3 Strategy for determining p95HER2 expression. CEER strategy for determining full length and truncated p95HER2 expression.
(TIF)

Figure S4 Disease-free survival differences between c-MET(+) HER1(−) vs c-MET(+) HER1(+) gastric cancer cohorts. Disease-free survival differences after curative surgery in all (HER2(+) and HER2(−)) gastric cancer samples comparing the HER1(−) c-MET(+) vs HER1(+) c-MET(+) cohorts. Median survival of the two cohorts in gastric cancer patients is 46.17 months (HER1(+) c-MET(+)) and 82.80 months (HER1(−) c-MET(+)). Sample numbers in each cohort, p-values and hazard ratios are indicated.
(TIF)

Table S1 HER2 & p95HER2 expression and biomarker phosphorylations in HER2(+) and HER2(−) GCs. This table shows the HER2 gene amplification status (‘1’ is gene amplified and ‘0’ is not gene amplified) and HER2 IHC scores (represented as ‘0’, ‘1’, ‘2’ or ‘3’) of HER2(+) and HER2(−) gastric cancer samples both based on gastric cancer (GCA) and breast cancer (BCA) scoring criteria. The histological subtypes of the gastric cancer samples are represented by color coding: intestinal type (yellow), diffuse type (blue), mixed type (pink), and not determined (white). The table also summarizes the p95HER2 expression status (‘1’ is p95(+), ‘0’ is p95(−), ND is not determined) and the phosphorylation status (‘1’ is phosphorylated and ‘0’ is not phosphorylated) of HER1, HER2, HER3, c-MET, IGF1R and PI3K signaling molecules in each sample. Respective CU cut-offs for each marker are indicated at the top of the respective columns.
(XLSX)
