# Supplementary figures and images for: A Novel Proteomics-Based Clinical Diagnostics Technology Identifies Heterogeneity in Activated Signaling Pathways in Gastric Cancers
Source: PLoS One. 2013 Jan 25;8(1):e54644. doi: 10.1371/journal.pone.0054644 (PMC3556044; doi:10.1371/journal.pone.0054644)

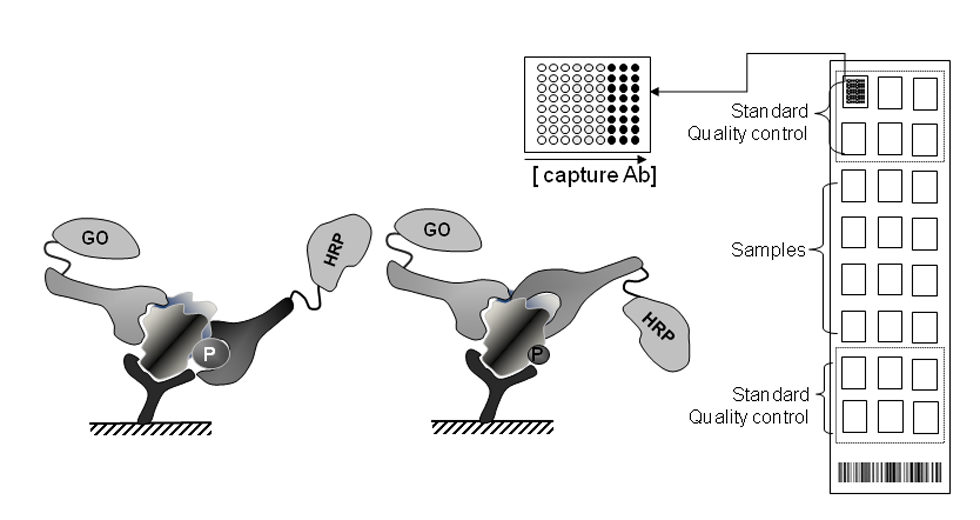

Supplement: Figure S1 — Principle of the CEER assay. Schematic showing the principle of the CEER assay and array layout. (Figure reproduced from Kim et al., 2011) (TIF) [file pone.0054644.s001.tif]

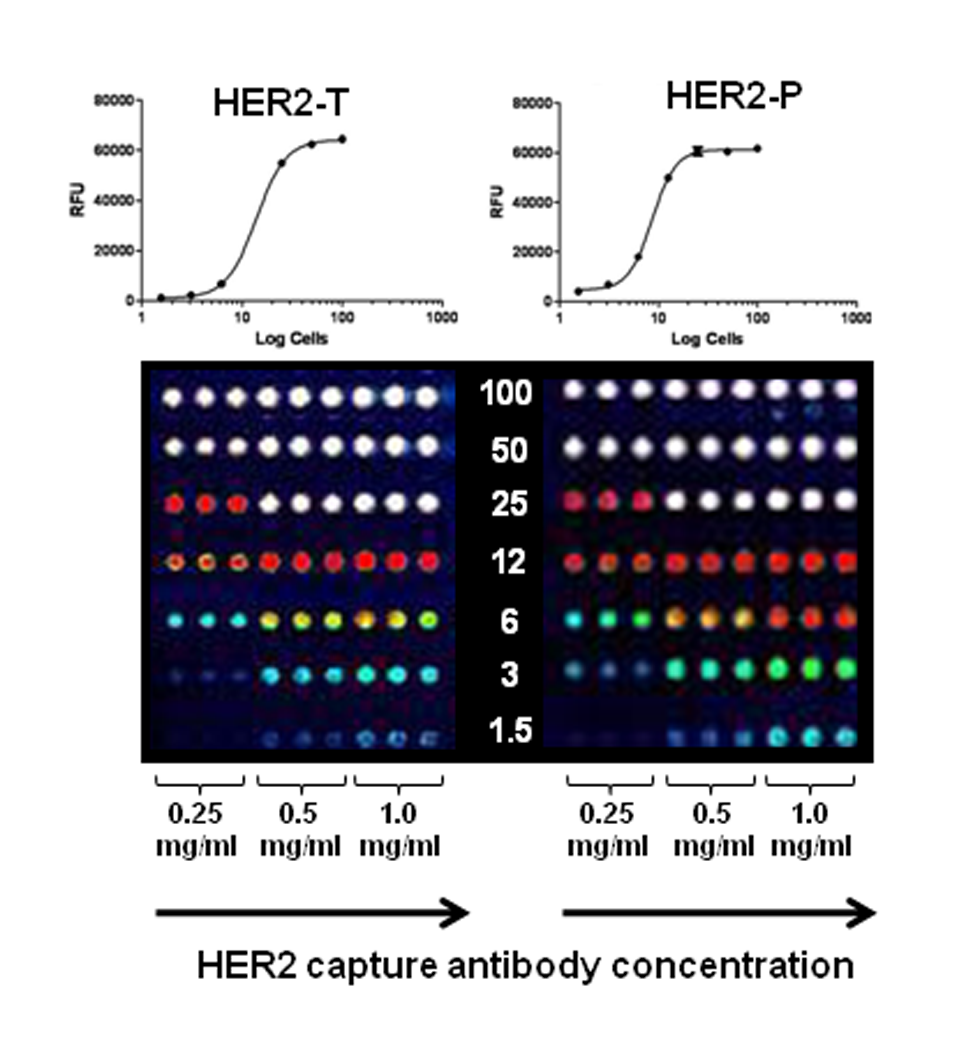

Supplement: Figure S2 — Standard curve for total HER2 and phosphorylated HER2. Standard curve of serially diluted cell lysates prepared from BT474 was used to normalize HER2 expression and the degree of phosphorylation in each sample. Each curve was plotted as a function of log signal intensity, measured as relative fluorescence unit (RFU) vs. log concentration of cell lysates and referenced to the standard cell lines. Image shown at single PMT setting, but multiple PMT scanning extends the dynamic range for the quantitation. Each row of the image shows total HER2 (HER2-T) and phosphorylated HER2 (HER2-P) expression in increasing numbers of cells. As shown, three different increasing concentrations of the HER2 capture antibodies were printed in triplicate on each CEER array. (TIF) [file pone.0054644.s002.tif]

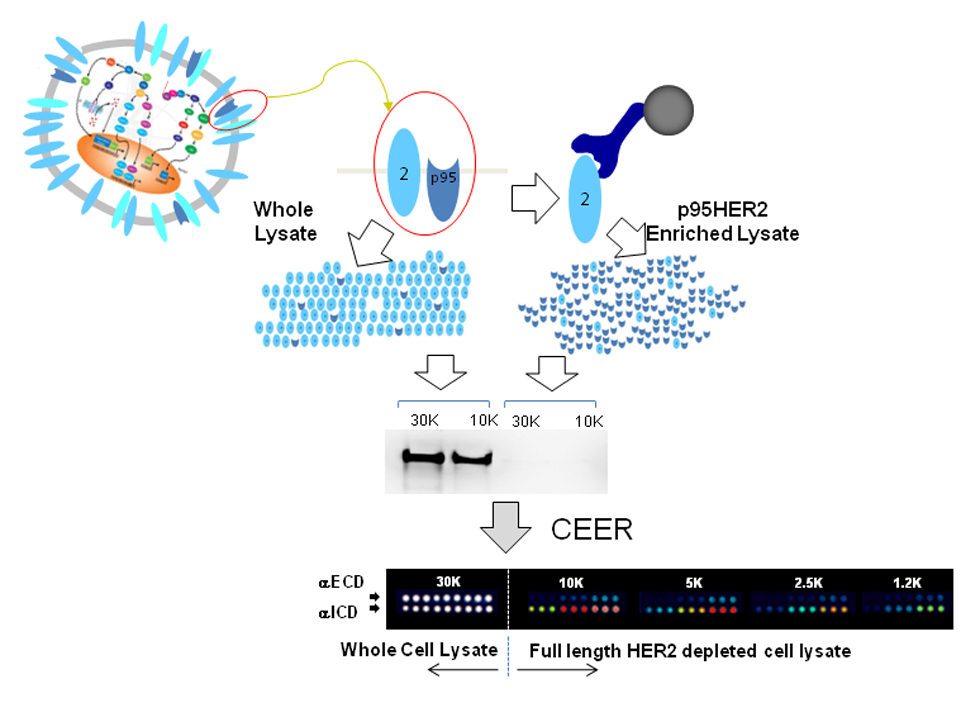

Supplement: Figure S3 — Strategy for determining p95HER2 expression. CEER strategy for determining full length and truncated p95HER2 expression. (TIF) [file pone.0054644.s003.tif]

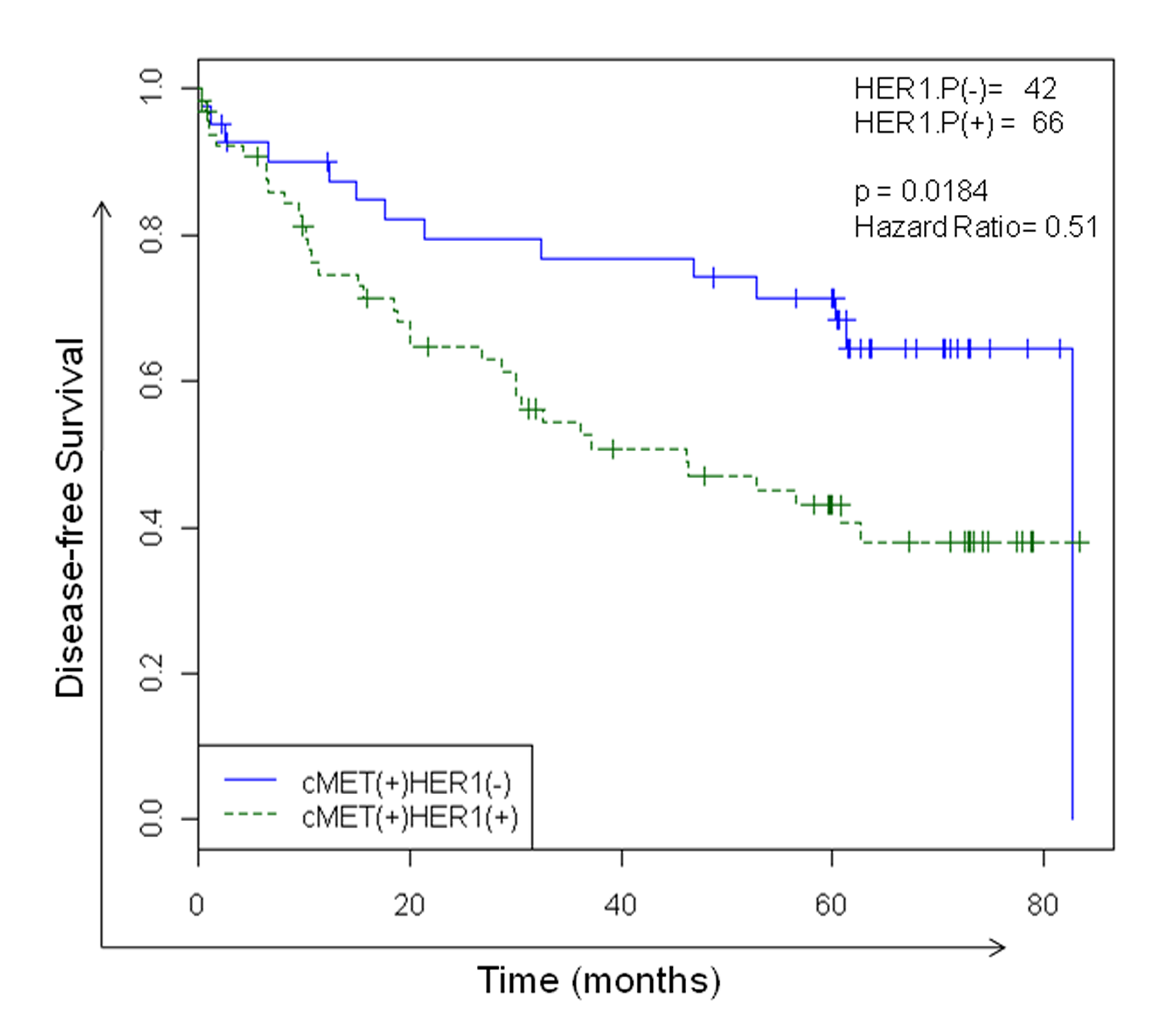

Supplement: Figure S4 — Disease-free survival differences between c-MET(+) HER1(−) vs c-MET(+) HER1(+) gastric cancer cohorts. Disease-free survival differences after curative surgery in all (HER2(+) and HER2(−)) gastric cancer samples comparing the HER1(−) c-MET(+) vs HER1(+) c-MET(+) cohorts. Median survival of the two cohorts in gastric cancer patients is 46.17 months (HER1(+) c-MET(+)) and 82.80 months (HER1(−) c-MET(+)). Sample numbers in each cohort, p-values and hazard ratios are indicated. (TIF) [file pone.0054644.s004.tif]
